# Explainable Artificial Intelligence (XAI) for Deep Learning Based Medical Imaging Classification

**DOI:** 10.3390/jimaging9090177

**Published:** 2023-08-30

**Authors:** Rawan Ghnemat, Sawsan Alodibat, Qasem Abu Al-Haija

**Affiliations:** 1 Department of Computer Science, Princess Sumaya University for Technology, Amman 11941, Jordan; 2 Department of Cybersecurity, Princess Sumaya University for Technology, Amman 11941, Jordan

**Keywords:** artificial intelligence (AI), explainable AI (XAI), deep learning (DL), convolutional neural network (CNN), medical imaging analysis, classification

## Abstract

Recently, deep learning has gained significant attention as a noteworthy division of artificial intelligence (AI) due to its high accuracy and versatile applications. However, one of the major challenges of AI is the need for more interpretability, commonly referred to as the black-box problem. In this study, we introduce an explainable AI model for medical image classification to enhance the interpretability of the decision-making process. Our approach is based on segmenting the images to provide a better understanding of how the AI model arrives at its results. We evaluated our model on five datasets, including the COVID-19 and Pneumonia Chest X-ray dataset, Chest X-ray (COVID-19 and Pneumonia), COVID-19 Image Dataset (COVID-19, Viral Pneumonia, Normal), and COVID-19 Radiography Database. We achieved testing and validation accuracy of 90.6% on a relatively small dataset of 6432 images. Our proposed model improved accuracy and reduced time complexity, making it more practical for medical diagnosis. Our approach offers a more interpretable and transparent AI model that can enhance the accuracy and efficiency of medical diagnosis.

## 1. Introduction

The World Health Organization (WHO) announced in March 2020 that the coronavirus outbreak, which had reached a life-threatening level, had spread to 58 nations worldwide [1]. One of the COVID-19 epidemic’s primary symptoms, initially reported by the WHO near the end of 2019, is a severe cough and difficulty breathing [2].

CT (computed tomography) is a cross-sectional imaging technique that uses a computer to interpret data obtained by quickly spinning an X-ray around the patient’s body [3]. Because it provides more comprehensive information than ordinary X-rays, chest X-ray (CXR) is frequently combined with CT to identify various diseases [4]. Because of their extensive use and high accuracy, deep learning models, also known as Artificial Intelligence (AI), have gained popularity over the last ten years [5]. Despite its numerous advantages, artificial intelligence (AI) presents obstacles at every medical sector research and deployment stage [6]. The main reason for these obstacles is that health-related systems require actual rather than synthetic data. As AI models become more complex, with hundreds of layers and millions of artificial neurons, the algorithms become less understandable [7].

**Figure 1:**
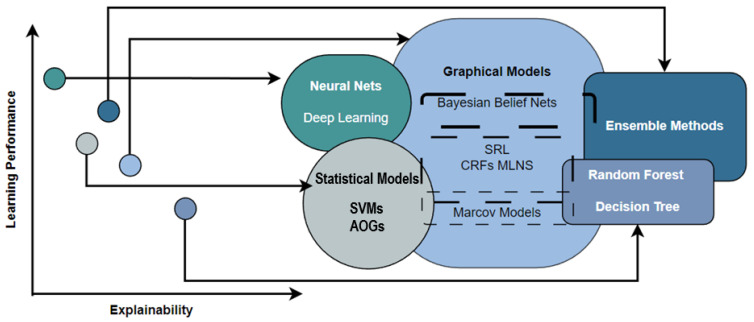
Artificial intelligence performance versus explainability [23].

In some instances, legal approval is contingent on the system’s comprehension. A great deal is published on black-box algorithms. As a result, there is a pressing demand for AI that can be explained [7]. The goal of “explainable AI” (XAI) is to be able to explain why AI systems exist, identify their capabilities and limitations, and predict how they will evolve in the future (see Figure 1 in the Literature Review section). It depicts the relationship between explainability and learning performance [8]. This relation brings a trade-off between explainable AI and high learning performance (typically measured by the accuracy representing the model’s performance) [9]. Without an auditing approach based on explainability [4], such cases of “right choice, wrong rationale” are challenging to track and discover. The decision emphasizes the necessity of explainability in improving the reliability of deep neural networks in clinical applications [10,11,12].

COVID-19 detection, for example, is a clinical use [7,13,14,15], so it is vital to create deep neural network architectures with transparency and accountability in mind, such as those predicted by this work’s conclusions. Furthermore, plotting and charts to clarify predictions can help explore and find relevant areas of categorization [16]. As a result, creating a multimodal neural network and integrating technical knowledge with understanding information to provide decision rules to enhance diagnosis fairness is essential [17]. As a result, we use an explainability approach to analyze how the model makes predictions. The goal is to obtain better insights into significant elements connected with COVID-19 cases. It can help clinicians to monitor better and evaluate the model. The evaluation will be transparent and accountable to maintain that it makes these decisions based on the related information from the CXR images, such as incorrect information represented outside the body, engrained markup representations, and image-processing objects. From the standpoint of explainability, AI applications are a vital and current subject of study for scientists [11]. The predictions offered by the proposed model become more transparent and trustworthy for physicians to employ throughout their screening method, allowing them to make faster but more accurate evaluations by pinpointing the main aspects [17].

Deep neural networks mainly provide findings that are sometimes difficult to comprehend [7]. This issue generated clarification calls for openness before using an algorithm for medical care [18]. The complexity of sophisticated applications grows in lockstep with their precision, making it more challenging to describe [19,20]. The proposed model’s critical components may help clinicians acquire unique knowledge of the primary visual signals correlated with the COVID-19 influenza virus, which they may utilize to improve screening accuracy. The method suggested to reach this goal is to create new or updated AI techniques that result in more explicable models. These models are available for users with cutting-edge human-computer interface approaches for clear and valuable explanation conversations for the end-user. This research applied CNN to the latest COVID-19 datasets to overcome the explainability limitation. Furthermore, we explained the type of results obtained by an AI model based on data sources and how they influenced the outcomes. In addition, the explainability feature of AI models is raised, especially in the clinician sector, due to its sensitivity for explaining causes and results. Finally, AI algorithms become more transparent (opposite to black-box algorithms) to the users.

### 1.1. Our Contribution

In this study, we provide the ‘XAI’ model, an explainable deep neural network approach for automatically detecting COVID-19 symptoms from CXR pictures. We intend to examine CXR images from COVID-19 instances. A series of methods for creating class-discriminating zones on the subjects’ chests were provided to achieve COVID-19 detection transparency. The purpose is to explain why the model prioritizes categorization in certain regions. Furthermore, the model will help explain, via data visualization, the difference between individuals suffering and those who are not. It will also aid in comprehending the COVID-19 elements. To describe less complicated models, the radar plot approach, for example, employs the weights of criteria, partial dependence plots, and individual conditional expectation plots. Finally, it will describe how a model creates predictions or relevant feature sets from the data as a decision process. Therefore, contrary to what was suggested, the ‘XAI’ paradigm will not replace radiologists [21]; it will be another option in a clinical situation rather than a replacement for a human radiologist. However, human judgment is necessary when patients’ lives are at stake [22].

Medical datasets have limited samples concerned with clinical usage. This article presents deep learning approaches to perform binary classification (as normal and COVID-19 cases) and multiclass classification (as COVID-19, pneumonia, and normal cases) of CXR images. Accuracy, precision, recall, loss, and area under the curve (AUC) are utilized to evaluate the performance of the models. This article also added a visual explanation to illustrate the basis of model classification and perception of COVID-19 in CXR images.

While our study’s deep learning models and explainer algorithms may have been established a few years ago, our work builds upon these foundations by introducing a novel ‘XAI’ model specifically designed for COVID-19 detection from chest X-ray (CXR) images. By adapting and applying existing deep learning architectures, we prioritize transparency and interpretability, essential for gaining insights into the model’s decision-making process. The use of the LIME algorithm further enhances the explainability of our approach by generating class-discriminating regions on CXR images, enabling healthcare professionals to understand why the model prioritizes certain regions during classification.

Our study used data visualization techniques to aid in the comprehension of COVID-19 elements and differentiate between individuals suffering from the disease and those who are not. Through radar plots, partial dependence plots, and individual conditional expectation plots, we comprehensively understand the model’s behavior and the factors contributing to COVID-19 detection. It is important to note that our ‘XAI’ paradigm does not aim to replace radiologists but serves as an additional tool in the clinical setting. We recognize that human judgment and expertise are critical, particularly when patients’ lives are at stake. The ‘XAI’ model complements the radiologist’s decision-making process by providing valuable insights and supporting diagnostic capabilities.

Furthermore, our study tackled the challenge of the limited availability of medical datasets by conducting binary and multiclass classification of chest X-ray (CXR) images for COVID-19 detection. This is a crucial contribution, as medical datasets are often constrained in size and diversity, especially in clinical usage. By designing and implementing deep learning approaches for binary (normal vs. COVID-19 cases) and multiclass (COVID-19, pneumonia, and normal cases) classification, we aimed to provide valuable insights into the detection and differentiation of COVID-19 from CXR images.

We employ rigorous evaluation metrics such as accuracy, precision, recall, loss, and area under the curve (AUC) to assess the performance of our models accurately. Additionally, our integration of visual explanations provides an intuitive and transparent basis for model classification and enhances the interpretability of deep learning models in the context of COVID-19 detection. By empowering radiologists with actionable insights, our work advances medical image analysis and paves the way for further research in explainable deep-learning methods for healthcare applications. The healthcare field can greatly benefit from developing transparent and interpretable models that can assist medical professionals in making accurate diagnoses, improving patient care, and saving lives.

### 1.2. Paper Organization

The rest of this paper is organized as follows. Section 2 introduces the literature review to revise the previous work. Section 3 presents this work’s methodology to specify the methods and datasets. It compromises dataset description, division of the datasets, experiment setup, and visualization. Section 4 projects the results of the experiments with analysis. Section 5 concludes with remarks, limitations, and future work.

## 2. Literature Review

From an explainability standpoint, intelligent systems are a significant and active subject of study for researchers [11]. In some instances, formal compliance is contingent on the system’s comprehension. Many used black-box algorithms, which indicate a trade-off between demonstrable AI and more incredible learning performance (accuracy) [23], as illustrated in Figure 1.

There is often a trade-off between the performance of artificial intelligence (AI) models and their explainability. High-performing AI models such as deep neural networks may be very accurate in their predictions or decisions, but their internal workings can be complex and difficult to understand [24,25]. On the other hand, simpler models that are easier to explain may need to be more accurate. For example, decision trees are simple and interpretable AI models that can be used for classification tasks. However, they may perform less well compared to more complex models such as deep neural networks, especially when dealing with large and complex datasets [26].

Researchers are developing techniques to provide high performance and explainability to address this trade-off. One such approach is the development of interpretable deep learning models, which use neural networks with additional constraints or architectures that make them more interpretable. Another approach is to use post-hoc explanation techniques, which analyze the decisions made by black-box models and provide explanations for them. Ultimately, the choice between performance and explainability depends on the specific application and its requirements. Explainability may be crucial for ensuring trust and accountability in some cases, such as medical diagnosis or legal decision-making. Performance may be more important than explainability in other cases, such as image or speech recognition [27].

In this section, we will briefly review the use of Artificial Intelligence models in COVID-19 detection as an essential medical case by referring to the literature and making a comparison summary to show the differentiation of this work.

In response to the need for quicker interpretation of radiography images, a range of deep learning-based artificial intelligence (AI) systems have been created [28]. Since the original release of the proposed COVIDx collection and the proposed COVID-Net, several studies on COVID-19 detection using CXR images have been conducted [29,30]. Many have undertaken comparable studies using COVIDx or COVID-Net versions [31]. As sophisticated applications improve accuracy, their networks have grown, making them hard to comprehend [7].

In a perfect scenario, we should anticipate the most significant explanations from a system that produces the best outcomes [32]. It eventually becomes an optimization problem, and it is vital to walk a fine line between great results and comprehensibility [17]. It is also helpful in detecting biases in datasets. Intelligent systems are crucial and present a research issue for researchers regarding interpretability [33].

The present research might Involve”appl’Ing natural language processing techniques to direct the psychiatrist’s patient information, making summaries from radiological pictures, or responding to visual inquiries in practice [33]. Based on the concentration of the characters in the layers of the deep convolution network-based model, it illustrates which sections of the picture information are utilized in classification tasks [34]. The supervised classification relationship of the first and deeper layers aids in analyzing complex neurological systems [32,35]. Furthermore, it is intriguing that too many aspects of medication administration are not considered. This is another scientific side of the debate with societal implications [36].

Wang et al. [7] used COVID-Net, a deep convolutional neural network, to identify COVID-19 in chest X-ray (CXR) pictures. A suggested COVIDx as an accessible benchmark dataset was displayed, consisting of 13,975 CXR images from 13,870 patient cases. Moreover, they had to use an explainability approach to analyze how COVID-Net concludes. It obtains valuable insights into significant COVID-19-related elements that can assist physicians in better oversight. It reviews COVID-Net transparently and credibly to verify that it is decided based on the information from the mentioned CXR images. The utilization of many heavy connections, such as in intensively deep neural network architectures, has the drawback of raising computation costs and storage expenses.

Open AI welcomes neuro-scientific specialists to provide explainability and interpretability [37]. As a result, there has been a study on the explainability of modeling using a machine-driven creative, experimental investigation. It assesses which portions of the photos were examined [4]. COVIDNet-CT, a deep convolutional neural network architecture, was shown to detect COVID-19 occurrences from chest CT images. In addition, they developed an explainability-driven efficiency verification approach to investigate COVIDNet-decision-making CT’s behavior, guaranteeing that COVIDNet-CT makes a prediction based on relevant indications in CT images for consistency and transparency. Performance verification is based on explainability to verify the claims on relevant image characteristics and to acquire a more profound knowledge of the CT image features. Nonetheless, developing solutions for the problem is contingent on qualified datasets.

The suggested explainable models keep changing when additional insights are incorporated and publicly revealed, as represented by a screenshot of the present state of the other models, and the accompanying XAI model that was denoted by [14]. It suggested an explainable deep neural network (DNN)-based approach for automated COVID-19 symptom recognition from CXR pictures. They examined 15,959 CXR images from 15,854 people, including normal, pneumonia, and COVID-19 cases. CXR images are thorough before being supplemented and classified with a neural ensemble method that employs gradient-guided class activation maps (Grad-CAM++) and layer-wise relevance propagation to emphasize category areas (LRP). It cannot, meanwhile, confer with physicians to validate diagnostics determined by the performance. Furthermore, successful assumptions are based not only on individual imaging modalities but also on multiple modes like CT and other critical factors like the patient’s socioeconomic and symptom assessment report.

Bhowal et al. in [17] used Ensemble learning to improve the classifier’s performance of deep learning techniques. They applied the Choquet method to aggregates using coalition game theory, information theory, and fuzzy lambda approximation and presented a novel approach for evaluating fuzzy measurements. They used three alternative scaling techniques, pattern recognition, and coalition game theory to construct the fuzzy measures. On the other hand, choosing helpful classifications from a group of categories that might or might not communicate important information necessitates experimentation. Computing marginal participation is unrealistic and sometimes unattainable due to the difficulty in determining the formulae for similarity measures and contingent correlation.

Zhong et al. in [12] developed a deep metric learning-based CXR image retrieval framework. Their proposed model uses a multi-similarity loss function, which helps train the model to identify similar images. At the same time, the hard-mining sampling strategy focuses on difficult examples to improve the model’s performance. The attention mechanism allows the model to identify disease-related regions within the images and provide useful visualizations of those areas. It produces similar pictures, representations of disease-related focus mappings, and essential clinical knowledge to guide treatment choices. The trained algorithm retrieves visual characteristics from a new dataset without further training. Nevertheless, there is a semantic gap between the information gathered from a photograph by algorithms and human interpretation of the same photograph.

Other approaches provided semi-supervised learning (SSL) strategies for learning with small amounts of data [19]. Researchers included local phase CXR image characteristics into a convolutional neural network architecture, training the SSL approach with a teacher/student paradigm. Statistical evaluation was performed on 8851 normal (healthy) CXR scan results, 6045 pneumonia images, and 3795 COVID-19 CXR scans. This proposed model can improve the accuracy of CXR image classification tasks, particularly with limited labeled data available for training. However, as with any machine learning model, it is essential to thoroughly validate its accuracy and generalizability before it can be widely adopted in clinical practice.

A new deep network for robust COVI”-19 recognition (MUL) by utilizing Deformable Mutual Information Maximization (DeIM), Mixture High-order Moment Feature (MHMF), and Multi-expert Uncertainty-aware Learning [10] to suggest RcoNetk DeIM reliably calculates and maximizes the similarity matrix (MI) between inputs and implicit interpretations to acquire concise and completely detached expressive features. Meanwhile, MHMF can thoroughly study the benefits of high-order analytics in medical imaging and identify discriminant information from complex ranges. Finally, for each CXR picture, MUL builds a slew of parallel dropout networks to assess uncertainty and, as a result, reduce performance degradation due to data noise. On the other hand, extremely high-order instances may reduce performance, which might be because all these properties are not helpful for COVID.

Qi et al. in [19] is an example where the authors built a one-of-a-kind multi-feature convolutional neural network (CNN) design for improved multiclass COVID-19 recognition from CXR images, and a local phase-based image enhancement technique was applied. The improved images and the original CXR data are fed into their suggested CNN model. They established the influence of more acceptable images on diagnostic accuracy using ablation trials.

Motamed et al. in [11] proposed a RANDGAN (randomized generative adversarial network) that distinguishes photographs of an unidentified class (COVID-19) from recognized and labeled classes (Normal and Viral Pneumonia) without using labeling or training the model from the unknown class of images (COVID-19). COVIDx, the most significant publicly available COVID-19 chest X-ray dataset, was utilized. It comprises images from several public databases and contains Normal Pneumonia and COVID-19. Transfer learning can distinguish the lungs in the COVIDx dataset. Moreover, they demonstrated why sectioning the region (lungs) is crucial for successfully learning the classification job, especially in datasets that include pictures from multiple resources, such as the COVIDx dataset. However, the separation model tends to be used in several cases.

Several data augmentation procedures increase the overall model performance of generalizing and resilience [38,39]. Conversely, research on microdata is just being achieved through combining AI research with clinical use. Small data is essential to provide important information, while vast data only survives with this base. We can translate AI into a slightly elevated, real-world medical application by merging little and large amounts of data [40,41]. It is possible to build an intelligent health and clinical services application by consistently mixing large and small data [42]. Nevertheless, due to the need for more sufficient and precise data on COVID-19, the deep learning work completed thus far cannot be deployed in institutions. Therefore, in many studies, the volume of data is a considerable constraint. The other goal of this study is to attain explainability AI by delving deeper into these datasets to construct more trustworthy, understandable, and visually appealing algorithms [43]. To overcome these limitations, we implement the model on different datasets. Moreover, XAI will outperform the ambiguity of AI algorithms.

The proposed explainable AI provides accurate predictions and clear and interpretable explanations of how those predictions were made. Table 1 summarizes related work. There have been many recent advances in X-CNNs for medical applications, focusing on developing models that can provide accurate predictions and meaningful explanations. Some related work in this area includes:

- Attention-based models: Attention-based models use an attention mechanism to highlight the input image’s regions most relevant to the output prediction. This can help provide visual explanations for the model’s predictions [7,41].
- Gradient-based methods: Gradient-based methods use the gradient of the model’s output concerning the input image to generate saliency maps that highlight regions of the image that are most important for the prediction. These maps can provide insights into which features the model is using to make its prediction [4,10,14].
- Model visualization: Model visualization techniques use optimization methods to generate images that maximize the activation of specific neurons or layers within the model. These images can provide insights into which features the model is sensitive to and how it is processing information [11,12,19].
- Rule-based models: Rule-based models use logical rules to generate explanations for the model’s predictions. These models can generate human-readable explanations that clinicians can easily understand [17].

The models presented In the related work significantly contribute to the models’ interpretability, reliability, and overall performance in medical imaging classification. The selection of these models was driven by the need for explainable predictions, allowing for insights into the reasoning behind certain predictions, particularly in critical decision-making scenarios. Deep Convolutional Neural Networks (CNNs) were chosen due to their ability to capture complex patterns and features in medical images, leading to enhanced prediction accuracy. However, it is important to consider the trade-off of increased computational complexity and memory cost associated with densely connected deep neural networks.

The Deep COVID Explainer model was selected to utilize a neural ensemble technique augmented and classed using Grad-CAM++ (gradient-guided class activation maps). This technique enables the model to generate explanations for its predictions by identifying and highlighting the important regions in the input image contributing to the predicted class. Such interpretability improves the understanding of the model’s decision-making process. It is worth noting that accurate predictions often necessitate a comprehensive approach beyond single imaging modalities.

Another selected model, COVIDNet-CT [4], incorporates an explainable artificial intelligence (XAI)-driven performance validation technique, leveraging XAI methods to validate and interpret the model’s performance. Conversely, RcoNetk [9] utilizes numerous parallel dropout networks to evaluate uncertainty. Uncertainty estimation plays a vital role in medical applications, providing insights into the confidence level of the model’s predictions. Parallel dropout networks in RcoNetk effectively assess uncertainty. However, it is crucial to ensure the availability of high-quality datasets during model training to achieve accurate uncertainty estimation.

The significance of segmented regions of interest in medical imaging classification is emphasized by RANDGAN [10], as accurate classification often relies on specific regions. Proper segmentation enhances feature capture and improves classification accuracy. Nevertheless, RANDGAN acknowledges that there may be circumstances where the segmentation model fails, warranting caution in utilizing this approach. Additionally, the Unique multi-feature CNN model showcases the impact of improved images on enhancing diagnosis accuracy through ablation trials. By systematically removing specific features or components from the model, the model identifies their contributions to diagnosis accuracy, improving overall performance.

Lastly, ensemble learning with deep learning was chosen due to its unique method for evaluating fuzzy measures. By combining the predictions of multiple models, ensemble learning enhances overall performance and robustness. However, it is essential to consider the increased computational complexity and memory requirements associated with training and combining multiple models.

Overall, there is growing research on X-CNNs for medical applications, with a focus on developing models that are not only accurate but also transparent and interpretable. The proposed model can change medical decision-making by providing clinicians with valuable insights into how predictions are made and helping build trust in machine learning systems using visualization. This study applied the explainable AI model to different datasets, including the COVID-19-image dataset, the COVID-19 and Pneumonia Chest X-ray dataset, and the Chest X-ray (COVID-19 and Pneumonia) dataset, in order to increase the interpretability of the AI model. By integrating LIME into an explainable AI model for medical image analysis, the model can identify areas for improvement or further decisions. This helps to improve the accuracy, reliability, and interpretability of AI systems for medical applications, ultimately leading to better patient outcomes.

In the proposed approach, LIME is used to visualize these interpretations and heat maps are used as a mask for the classified images. These heat maps highlight the specific areas of an image that contributed most to the model’s classification decision. This allows clinicians to understand how the model arrived at its diagnosis, which can be valuable in medical diagnosis using images.

The block diagram in the statement can be used as a template for implementing LIME in any medical diagnosis using images. By using LIME to interpret the results of machine learning models, clinicians can better understand how the model makes its diagnoses and make more informed decisions about patient care. In this work, we improved the model’s performance in terms of both time complexity and accuracy. Using the explainable AI model, we obtained more interpretable results, which can help clinicians better understand how the model is making its diagnoses. The model achieved an accuracy rate of 90.6% on a relatively small dataset of 6432 images.

## 3. Methodology

**Figure 2:**
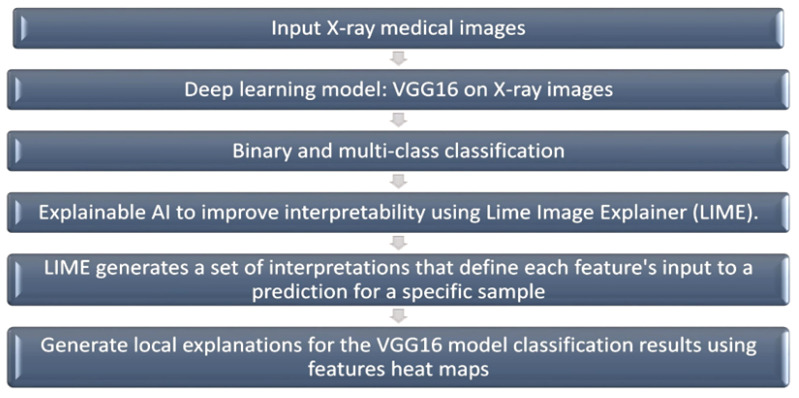
Block diagram for the proposed model (VGG16 with LIME).

The main contribution of this work is enhancing the deep learning approaches in healthcare applications using the visualization capability of explainable AI, which is shown in the block diagram in Figure 2. We started from having the medical images and then we inputted those images into the deep convolutional neural network (VGG-16), which produces the binary classification of normal and COVID-19 cases and the multiclass classification of COVID-19, pneumonia, and normal cases. The explainable AI model that will interpret this result is Lime Image Explainer (LIME). LIME generates a set of interpretations that define each feature’s input to a prediction for a specific sample, which is a local understanding. Finally, we used visualization for the heat maps as a mask for the classified images to mark boundaries in the classification decision. This block diagram can be used in any medical diagnosis that uses images.

### 3.1. Datasets Description

This paper uses five CXR image datasets that are freely available on Kaggle. We selected CXR vertical and medical images in our study because radiologists typically use this radiography component to complete diagnostic imaging assessments.

The first dataset contains 5856 images. There were 1583 normal images and 4273 pneumonia images. In total, 10% of the CXR images in the dataset have been used for testing. The rest of the samples are divided between training and validation sets. Thus, the number of test images equals 624, while the training and validation image numbers are 5216 and 16, respectively. The test images have been randomly selected. Table 2 shows the division of the first dataset.

**Figure 3:**
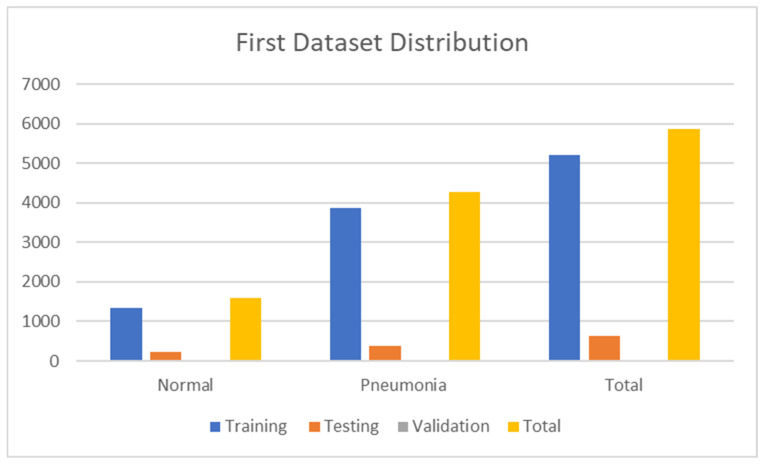
First dataset distribution.

Figure 3 also shows the numerical data of the first dataset. The dataset can be accessed at https://www.kaggle.com/paultimothymooney/chest-xray-pneumonia (accessed on 22 March 2023).

**Figure 4:**
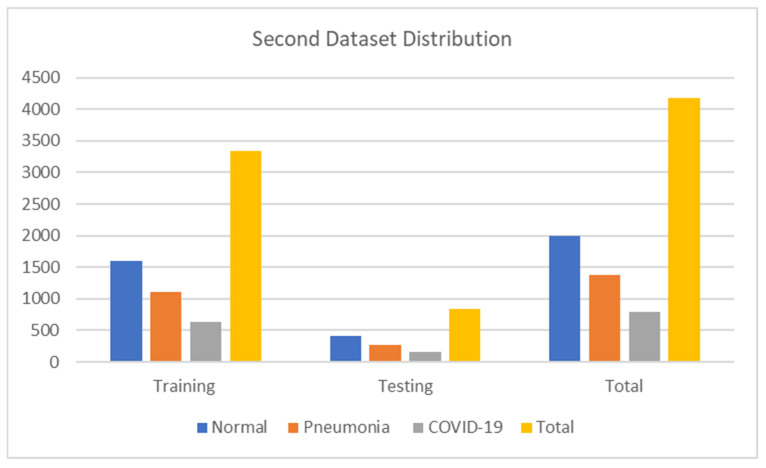
Second dataset distribution.

The second dataset contains 4172 images encompassing 2000 normal, 1380 pneumonia, and 792 COVID-19 images. Moreover, we have 3332 images for training, 840 for testing, and validation for training. The details are shown in Table 3. Figure 4 also shows the numerical data of the second dataset. This dataset is publicly available at https://www.kaggle.com/lelpresidente/covid19-and-pneumonia-chest-xrays dataset (accessed on 22 March 2023).

**Figure 5:**
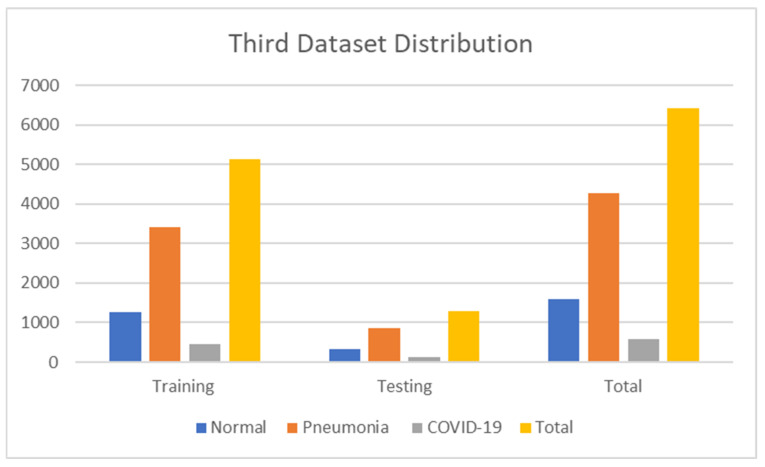
Third dataset distribution.

The third dataset contains 6432 images encompassing 1583 normal, 4273 pneumonia, and 576 COVID-19 images. Moreover, we have 5144 images for training, 1288 images for testing, and validation taken for training. The details are shown in Table 4. Figure 5 also shows the numerical data of the third dataset. The dataset is accessible at https://www.kaggle.com/prashant268/chest-xray-covid19-pneumonia (accessed on 22 March 2023).

**Figure 6:**
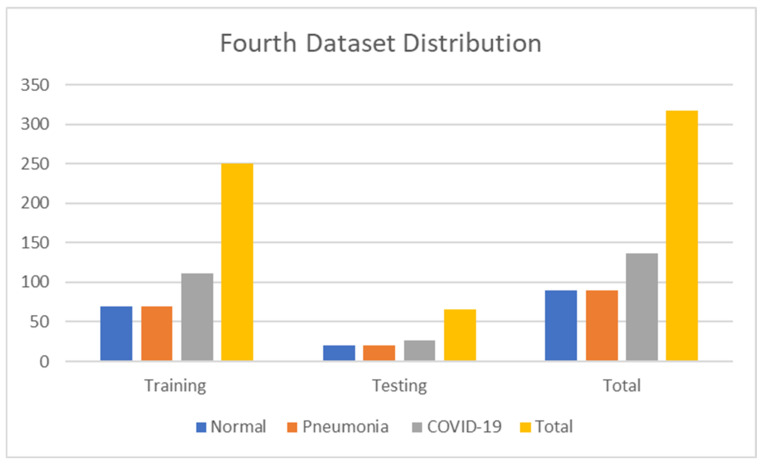
Fourth dataset distribution.

The fourth dataset contains 317 normal images, 4273 pneumonia, and 576 COVID-19 images. Moreover, we have 251 images for training, 66 images for testing, and validation taken for training. The details are shown in Table 5. Figure 6 also shows the numerical data of the fourth dataset. The dataset is accessible at https://www.kaggle.com/datasets/pranavraikokte/covid19-image-dataset (accessed on 22 March 2023).

**Figure 7:**
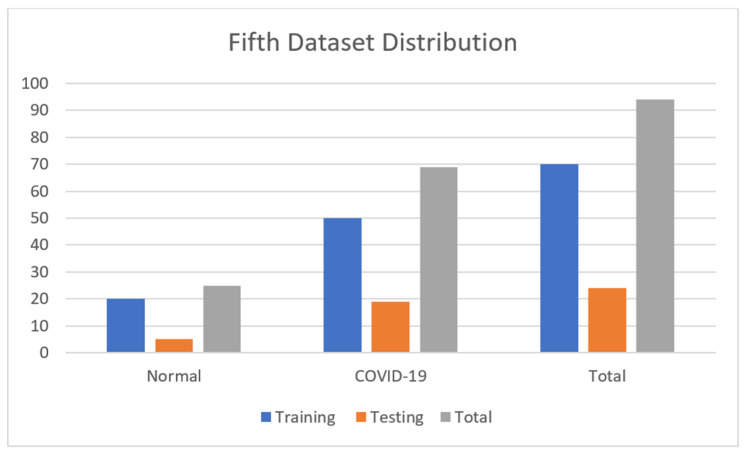
Fifth dataset distribution.

The fifth dataset contains 94 images encompassing 25 normal and 69 COVID-19 images. Moreover, we have 70 images for training, 24 images for testing, and validation taken for training. The details are shown in Table 6. The dataset is accessible at https://www.kaggle.com/datasets/alifrahman/covid19-chest-xray-image-dataset (accessed on 22 March 2023). Figure 7 also shows the numerical data of the fifth dataset.

### 3.2. Experimental Setup

We have used the Python programming language with the Keras package with Tensorflow as the deep learning framework to implement the proposed method. We run the codes on the Kaggle notebook with the following system specifications: Nvidia Tesla (NVIDIA Corporation, London, UK) T4 with 13 GB GPU memory. The software stack consists of scikit-learn and Keras with the TensorFlow backend. The model was trained using an Adam optimizer, sparse categorical cross-entropy loss function, a learning rate of 0.001 for the first epoch, and a learning rate decay of 0.1 every ten epochs with mini-batches of size 32. We have used VGG16 with input shape 224 × 224 × 3 with Dropout 0.5 and activation function Softmax. TensorFlow binary was optimized with one API Deep Neural Network Library (one) to use AVX2 AVX512F FMA CPU instructions in performance-critical operations. TensorFlow was rebuilt with the appropriate compiler flags to enable them in other operations. The GPU was used with 15,403 MB memory.

### 3.3. Visualization

**Figure 8:**
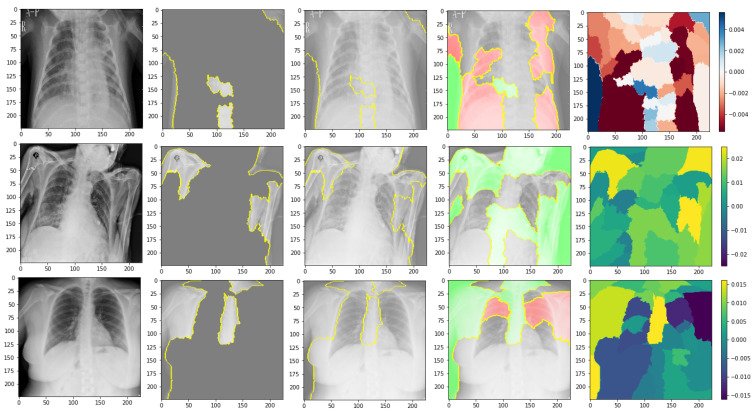
Explainability of X-ray images in the datasets’ examples of heat maps.

In this part, we use explainable AI to improve the interpretability of the COVID-19 analysis to overcome the black-box problem. It makes deep learning model predictions logical and intelligible in CAD-based COVID-19 diagnosis. We employed Lime Image Explainer (LIME). Local Interpretable Model-Agnostic Interpretations are a method for adequately explaining the predictions of any classifier or regressor. It approximates them locally using an interpretable model to modify a single data sample’s feature values and assesses the effect on the outcome. An “explainer” outlines estimates based on each sample data. LIME generates a set of interpretations that define each feature’s input to a prediction for a specific sample, which is a local understandability, as shown in Figure 8.

Using LIME, quickly understood models are regression analyses or decision trees learned on minor disturbances of the previous design (best areas, noise, removing words, and hiding areas of the image) to create a good local approximation. We used heat maps to obtain the image and mask function to mark boundaries because the visualization makes more sense if a symmetrical color bar is used. Each test X-ray image generates a heat map. Because there are several layers and filters, the averages of the weights of the filters in the final convolutional layer are computed and shown because they could directly represent the feature maps.

Every chest X-ray image has a heat map calculated to highlight high-weight COVID-19 signals. To create the last heat map, the weights of the filters are taken from the previous convolutional layer. Figure 3 displays the sample filter weights of a single chest X-ray image. In this illustration, the lungs’ areas are surrounded by high weights (yellow hue) since COVID-19 may harm the lungs. The weights of these filters are then averaged to obtain the final heat map. It is calculated for each test subject’s chest X-ray image. In Figure 3, examples of heat maps are displayed. The first three heat maps were generated using COVID-19 from chest X-ray scans. As can be observed, the trained model identified areas with significant weights of yellow spots as COVID-19 signal locations. Whereas the last heat map is generated from a chest X-ray image with the standard classification, the medical specialists would be focused on these areas to check the ailment finally. As a result, no yellow spots signify any COVID-19 harm.

Deep learning uses numerous hidden layers piled on top of one another. In addition to computer vision, deep learning has ushered in a new era of machine learning. CNNs have been used for object identification, segmentation, and classification of images [44]. Despite recent developments, we are still extremely early in the process. We have yet to decide on the optimal practices for network architecture in terms of deep design, compact size, and quick training [45].

Machine learning (ML) applications are becoming more prevalent and are being used to make a pathological diagnosis of various illnesses in the field of medical imaging. Computer-aided diagnostic systems have emerged due to several investigations [5]. Although there are numerous domains where image recognition has been used, medical images are one of them. Recent deep-learning advances in image recognition have sparked strong research interest in medical image segmentation [46].

The performance of classical image processing methods for image segmentation is no longer comparable to that of neural network (NN)-based approaches due to recent advances in deep learning and machine learning. As a result, several researchers have suggested enhanced deep learning algorithms to boost image segmentation precision in various recognition settings. The most popular method for recognizing images is CNN, which increases hidden layer depth and successfully acquires additional identifying features to increase segmentation accuracy [15]. Face object and license plate identification are successful image recognition applications. Medical image recognition is still less prevalent due to challenges in obtaining medical photos and a need for knowledge about how illnesses manifest in diverse images. Therefore, before moving on to model training, medical image recognition typically requires the help of a doctor to identify and classify focus regions or lesions [35].

The layered structure, set up in a tiered system, is the primary characteristic of deep learning approaches. Low-level details like textures and edges are extracted from the layers closest to the input [37]. Each layer’s feature extraction becomes more complex, and the acquired low-level characteristics are combined to create a more complex representation. CNNs are the most popular among the different deep-learning techniques since they can extract meaningful information from an image [41].

The input is typically loaded as a multidimensional vector and distributed to the hidden layers by the input layer. The learning process begins when the hidden layers consider the judgments made by the preceding layer and determine if a stochastic change inside itself worsens or enhances the output [42]. The weights of just one neuron of the first layer significantly rise when considering a more extensive colored picture input. Consider that the network must also be much larger than the one used to categorize color-normalized areas to handle this input scale. You will see the disadvantages of such models [44].

First, we implemented the VGG16 model to achieve prediction results. VGG is a convolutional neural network model that Simoyan and Zisserman proposed. They presented it in [45] as “extremely deep convolutional networks for large-scale image recognition”. Because the multilayer nonlinear layer may enhance the network depth to ensure learning more complicated patterns at a relatively low cost, the small convolution kernel is preferable to the big convolution kernel (fewer parameters). However, VGG employs more parameters and demands more processing resources, which increases memory use [46]. The first wholly linked layer, one of the three in the VGG-16, provides the most parameters. VGG16 can be used to identify illness via radiography, such as X-rays. VGG16’s potential has yet to be thoroughly investigated, although it performs incredibly well during image segmentation scenarios [47].

In this section, a description of the proposed method shows the architecture of the model and the methods used to achieve XAI. Although there is no specific aim for deep learning that involves simulation, there are various ways inspired by or based on neuroscience. Convolutional Neural Networks (CNNs) are the deep neural network method that has restored faith in ANN methods and found various applications, even though some have failed. The images in the experimental dataset are X-ray images; hence, multiple feature extraction and additional parameters are not required. To ensure that the model’s feature extraction is accurate, as well as to realize the model’s lightweight design and accelerate the model’s training, we will combine the original VGG-16 with the full convolution model and reduce the model’s parameters as well as the number of layers in the entire connection layer.

**Figure 9:**
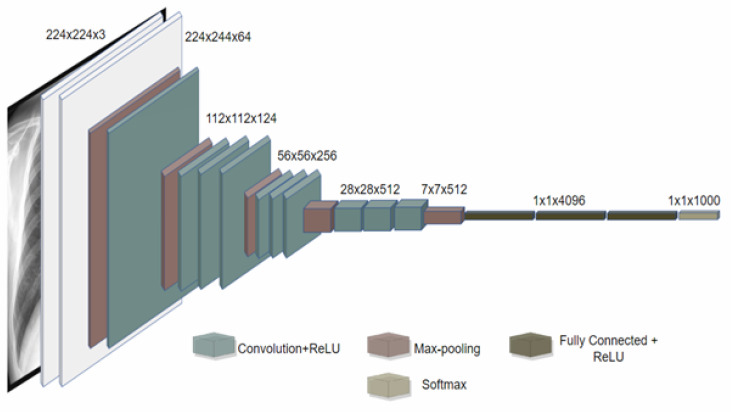
The architecture of VGG16 on X-ray images [5].

The convolution one layer receives a 224 by 224 RGB picture with a constant size as input. The image is processed using a stacking of convolutional (Conv.) layers with a tiny input patch: three-by-three (the lowest amount representing the concepts of moved near and middle). Inside one of the settings, it employs eleven convolutional filterings, regarded as a linear change of the input streams (followed by nonlinearity). The convolutional duration is set to one pixel, and the spatial pad to Conv. For a three-by-three VGG, the layer input is adjusted to one pixel layer to preserve the number of pixels upon convolution. The architecture depicted in Figure 9 is VGG16 [5].

Spatial pools perform five max-pooling layers that follow a portion of the Conv. Layers (not all Conv) are followed by max-pooling, and stride two is used to max-pool over a two-by-two pixel frame. With a stacking of convolution layers (ranging in an architecture), three fully connected (FC) layers are introduced: the first two have 4096 windows per, whereas the third provides 1000-way ILSVRC classification, and so has 1000 channels (one for each class). The top level is the soft-max layer. The entirely linked layers are constructed in the same manner in all connections.

In this work, we have accomplished implementing the VGG16 CXR image dataset to predict the presence of COVID-19 and to explain the results by showing the segments and colors in the images that aid in correct classification. We will implement the model on different datasets to ensure the model’s reliability according to investigating these datasets. We hope this will lead to new emerging techniques in adopting XAI models in COVID-19 prediction and diagnosis.

## 4. Experimental Results and Analysis

This section presents a discussion about the implemented XAI model’s results. We can apply the code to any dataset of COVID-19 X-ray images regardless of whether its method involves only training and testing or training, testing, and validation. Furthermore, it can run on datasets with any number of classes, either binary classes (COVID-19 and non-COVID-19) or multiple classes (COVID-19, normal, and pneumonia). The suggested technique allows for the initial classification classifier to categorize a chest X-ray image into COVID-19 and non-COVID-19. The non-COVID-19 class’s training and validation samples are normal chest X-ray images. It is primarily due to the training procedure revealing COVID-19 and regular case patterns. Its performance drastically reduces when it evaluates chest X-ray images with additional illnesses.

The importance of training and accuracy should pay attention to the focus on explainability in research papers. While improving model explainability is valuable, it should be achieved after establishing a robust and accurate model. The level of explainability depends on the domain-specific requirements, and there may be a trade-off between accuracy and explainability. Achieving the right balance is essential, considering the context and problem being addressed. For comprehensive results from all datasets, we provide all datasets’ validation loss and accuracy that reached ten epochs as given in Appendix A.

The decision to limit training epochs was intentional in our study on XAI for medical imaging classification. Our focus was on model interpretability rather than achieving complete convergence. We balanced model performance and practical considerations, ensuring meaningful representations and reasonable accuracy within a reasonable training timeframe. Extending training epochs would not significantly contribute to our primary objective of exploring model explainability.

**Figure 10:**
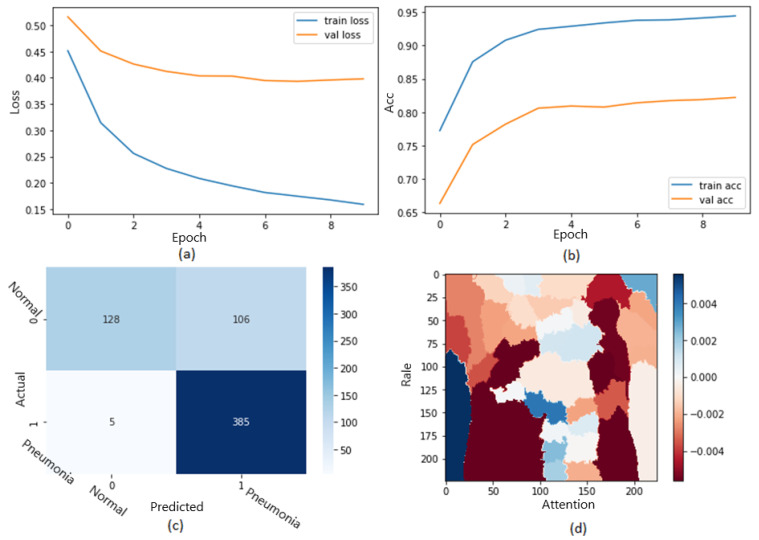
The results of the first dataset (loss in (**a**) accuracy in (**b**), confusion matrix in (**c**), and Attention map in (**d**)).

Figure 10 shows the ROC curve of the results of the first dataset (in Figure 10a,b loss and accuracy). Training and validation loss decreases to zero, while the training and validation accuracy increases to 82.6%. The confusion matrix is presented in Figure 10c,d as the heatmap scale. We might see a gap between training and testing accuracy in this dataset. This might refer to the small size of the validation dataset. As shown in Figure 10c, the cases of TP and TN explainability are presented (TP and TN samples utilizing LIME to locate COVID-19, pneumonia, and expected areas with CXR images). The heat map (Figure 10d) indicates the critical locations in the CXR images that our deep learning algorithm discovered.

**Figure 11:**
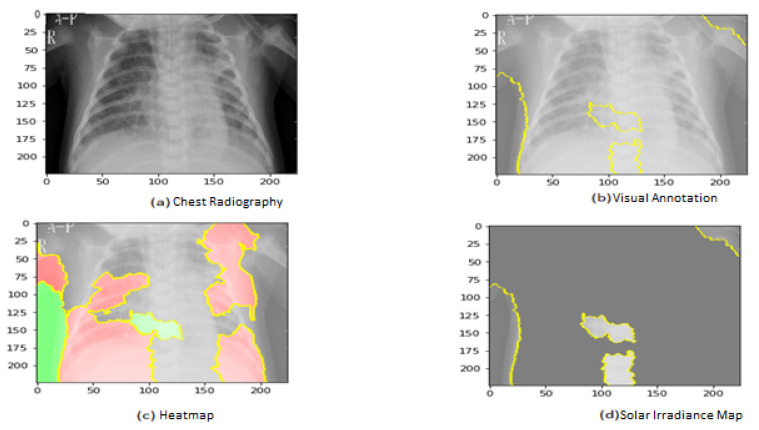
Explainability of X-ray images in the first dataset.

Figure 11 shows the explainability of one of the images in the first dataset, explaining the image segments that aid classification. Figure 11 also depicts the heatmap localizing indications in the lungs. Figure 11 shows an example of genuine positive instances from the COVID-19 dataset. The yellow color in the lungs implies that the model recognized something odd, thus classifying them as COVID-19. The model recognized the dense homogeneous opacity patches as the most significant COVID-19 signal, which fits well with radiology findings in COVID-19 medical research investigations [4,7,10,11,12,17,19].

**Figure 12:**
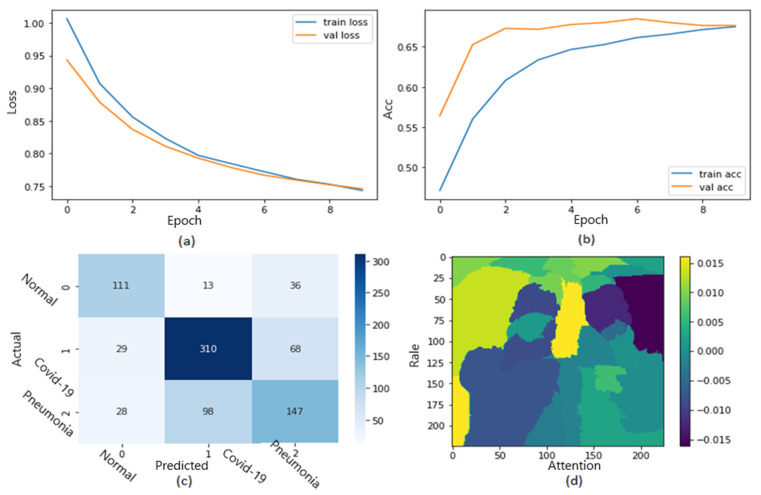
The results of the second dataset (loss in (**a**) accuracy in (**b**), confusion matrix in (**c**), and Attention map in (**d**)).

Figure 12 shows the ROC curve of the results of the second dataset (loss in Figure 12a and accuracy in Figure 12b).

Training and validation loss decreases to zero, while the training and validation accuracy increases to 67.5%. The confusion matrix is presented in Figure 12c,d as the heat map scale. According to the results, we must balance the testing dataset (especially COVID-19 cases) to obtain more accurate results. Additional TP instances of pneumonia samples are shown in Figure 12c for the second dataset. Similar to Figure 11, we can examine the positive pneumonia cases in the second dataset, with the heatmap focusing on the lung opacity area. Figure 12d displays the TN cases for the second dataset. The heatmap often concentrates on anything beyond the lungs (or close to the heart) to discriminate between the typical and other situations.

**Figure 13:**
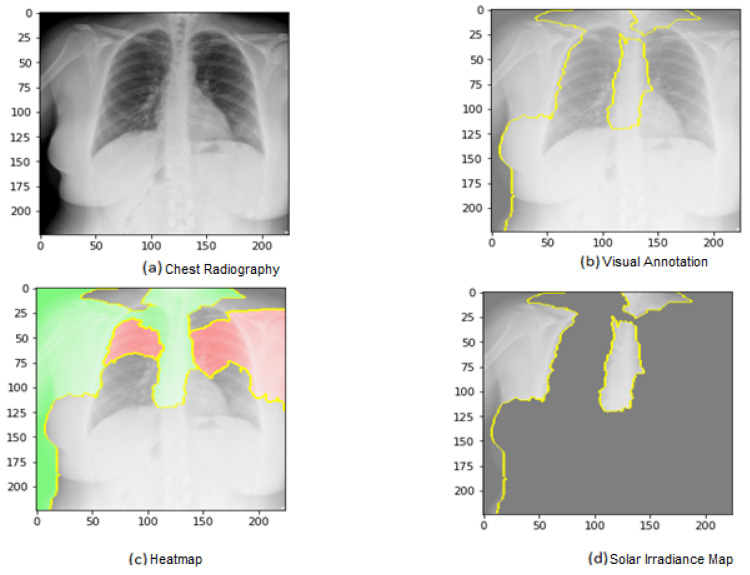
Explainability of X-ray images in the second dataset.

Figure 13 shows the explainability of one of the images in the second dataset, explaining the image segments that aid classification. We can observe in the CXR images that the heatmap identifies the density in the lungs. Despite the low quality and inaccurate projection of the lungs, the model accurately identified the images. The yellow color in the lungs implies that the model recognized something odd, thus classifying them as COVID-19.

We are shedding light on the specific image segments contributing to the classification process. The generated heatmap in the chest X-ray (CXR) images effectively identifies density areas within the lungs. Despite potential challenges such as low image quality and inaccurate lung projections, the model accurately identifies COVID-19 cases. The yellow in the lung regions indicates that the model has detected abnormal patterns, leading to the classification of these images as COVID-19. These findings demonstrate the model’s capability to discern subtle features and patterns in CXR images, even in challenging factors. As identified by the model, these regions hold valuable information that can provide a deeper understanding of the underlying pathological processes and aid in accurate disease classification.

For instance, in the context of chest X-ray (CXR) images, the model-generated heatmap highlights density areas within the lungs, indicating various pulmonary conditions. Understanding the significance of these high-weight regions can help healthcare professionals identify specific radiographic patterns associated with different diseases, including COVID-19. Furthermore, exploring the clinical implications of these regions can shed light on the anatomical and pathological characteristics relevant to disease diagnosis and treatment. Many medical insights can be derived from these regions; for example, clinicians can refine their diagnostic approach, develop targeted treatment strategies, and potentially uncover novel biomarkers or imaging markers for improved patient management. Incorporating these discussions into the evaluation and interpretation of model outputs enhances the clinical applicability and value of the model.

**Figure 14:**
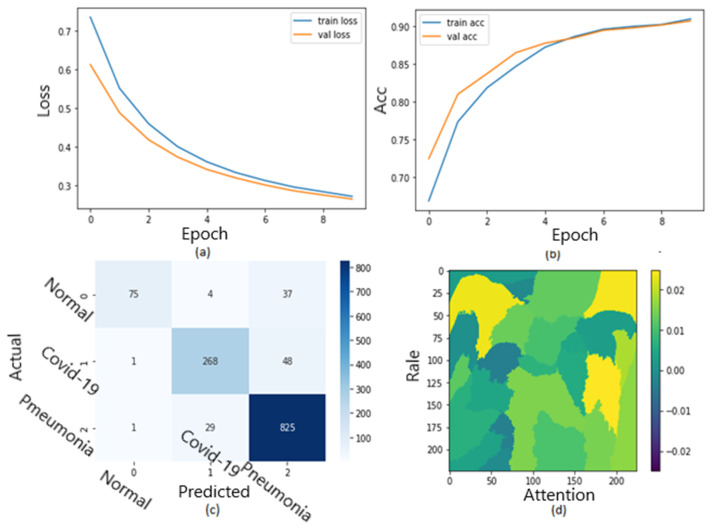
The results of the third dataset (loss in (**a**) accuracy in (**b**), confusion matrix in (**c**), and Attention map in (**d**)).

Figure 14 shows the ROC curve of the results of the third dataset (in Figure 14a,b sections). The confusion matrix is presented in Figure 14c,d as the heat map scale. Figure 14c shows the confusion matrix of the third dataset for the multiclass classification. Similar to the second dataset, the model provides a superior classification of the three types, as shown by the fact that only 30 patients out of 855 in the pneumonia class had incorrect classifications (see Figure 14c). According to the confusion matrix, the COVID-19 cases are accurately identified. It demonstrates how well our model performed in classifying the three classes. Both training and validation loss decrease to zero, while the training and validation accuracy increase to 90.6%.

**Figure 15:**
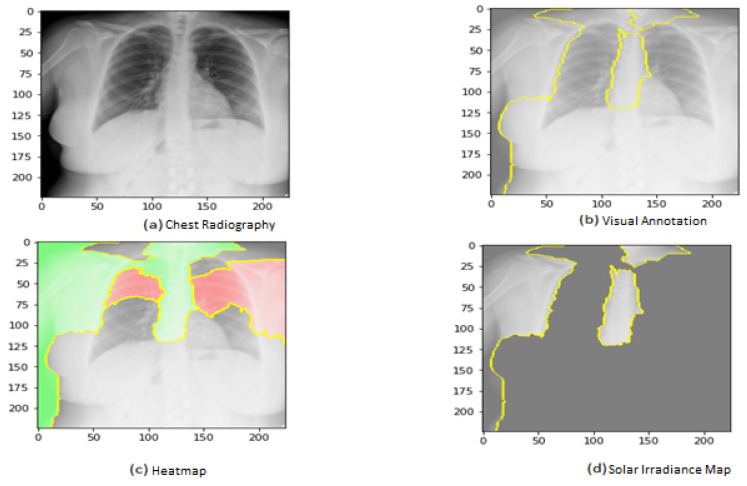
Explainability of X-ray images in the third dataset.

Figure 15 shows the explainability of one of the images in the third dataset, explaining the image segments that aid classification. As shown in the figure, Figure 15a represents the original image. In Figure 15b, the main segments that lead to classifying the image are outlined to show the importance of these features. Net, while Figure 15c represents only the important segments or features, the rest of the features were ignored in the last image. Figure 15 also displays the heat map localized to the points in the lungs. It is an example of genuine positive instances from the COVID-19 dataset. The yellow color in the lungs implies that the model recognized something odd, thus classifying them as COVID-19.

According to the expert, the produced heat maps of the COVID-19 instances successfully identified the locations of COVID-19. The use of heat maps of an image applied by our model appears when there are multiple foreign objects to be detected outwards. The lungs have yet to be annotated with a bounding box in the third dataset (best viewed, zoomed in color) from left to right.

**Figure 16:**
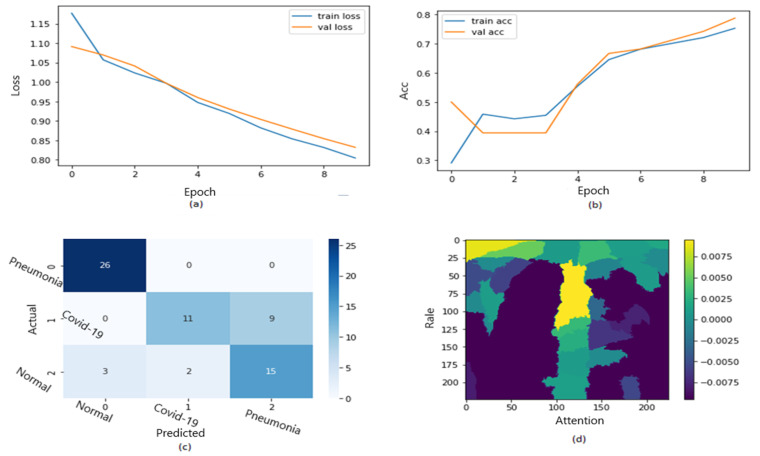
The results of the fourth dataset (loss in (**a**) accuracy in (**b**), confusion matrix in (**c**), and Attention map in (**d**)).

In Figure 16, the ROC curve for the fourth dataset is displayed in Figure 16a,b, showcasing the loss and accuracy. Both training and validation loss decrease to zero, while the training and validation accuracy increase to 95.7%. The confusion matrix is presented in Figure 16c and the heatmap scale is presented in Figure 16d. Figure 16c demonstrates the explainability of TP and TN cases, utilizing LIME to identify COVID-19, pneumonia, and expected areas in CXR images. The heatmap in Figure 16d highlights the crucial locations in the CXR images that our deep learning algorithm has detected.

**Figure 17:**
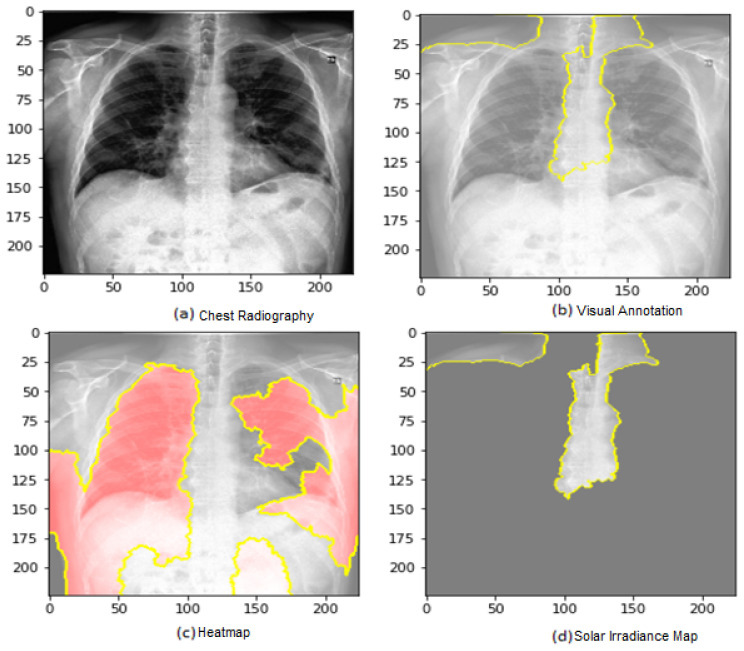
Explainability of X-ray images in the fourth dataset.

Figure 17 presents the explainability of an image in the fourth dataset, highlighting the image segments that contribute to the classification. Similarly, Figure 17 displays a heatmap that localizes indications in the lungs. Figure 17 showcases an example of genuine positive instances from the COVID-19 dataset, where the yellow color in the lungs suggests that the model has identified anomalous features, classifying them as COVID-19. Together, these figures demonstrate how our deep learning algorithm can identify critical features and patterns in CXR images, providing a potentially valuable tool for diagnosing COVID-19 and other respiratory diseases.

**Figure 18:**
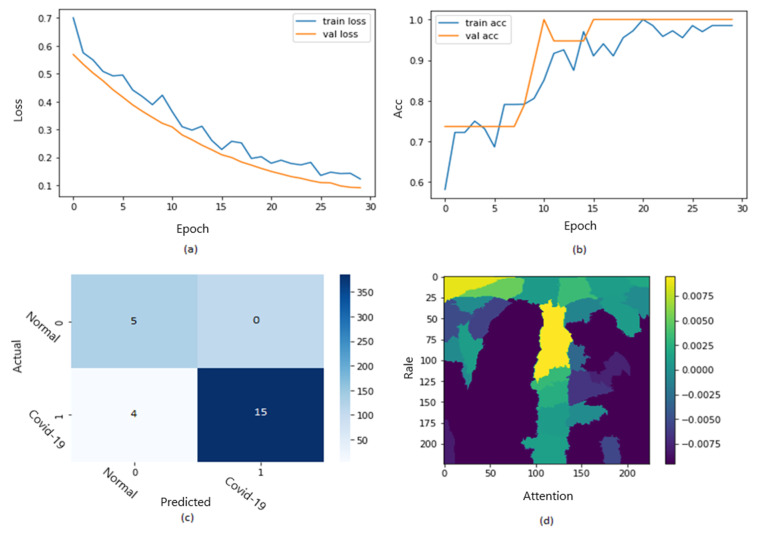
The results of the fifth dataset (loss in (**a**) accuracy in (**b**), confusion matrix in (**c**), and Attention map in (**d**)).

In Figure 18, the ROC curve for the fifth dataset is displayed in Figure 18a,b, showcasing the loss and accuracy. Both training and validation loss decrease to zero, while the training and validation accuracy increase to 93.7%. The confusion matrix is presented in Figure 18c and the heatmap scale is presented in Figure 18d. However, there appears to be a disparity between training and testing accuracy in this dataset, which may be attributed to the small size of the validation dataset. Figure 18c demonstrates the explainability of TP and TN cases, utilizing LIME to identify COVID-19, pneumonia, and expected areas in CXR images. The heatmap in Figure 18d highlights the crucial locations in the CXR images that our deep learning algorithm has detected.

**Figure 19:**
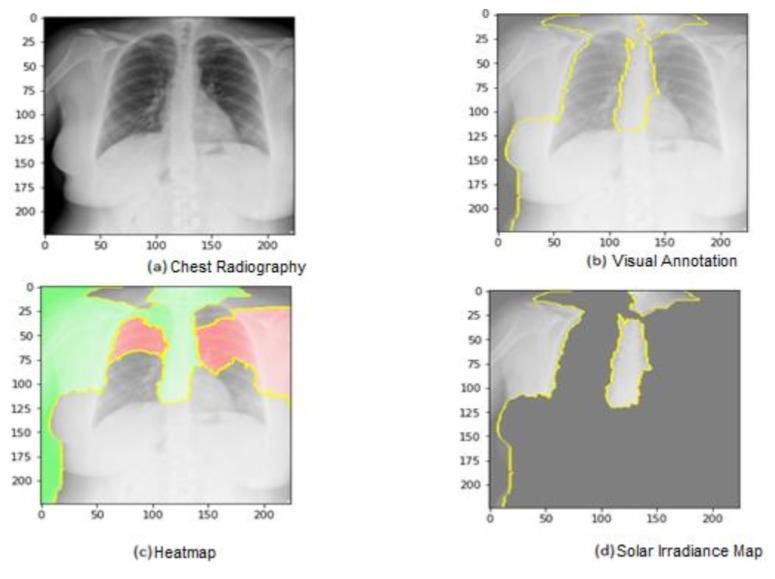
Explainability of X-ray images in the fifth dataset.

One of the images from the fifth dataset is displayed in Figure 19, which illustrates the image segments that assist in its classification, explaining its explainability. Additionally, Figure 19 demonstrates a heatmap that identifies indications in the lungs. Figure 19 showcases authentic positive instances from the COVID-19 dataset, with the yellow coloring in the lungs indicating that the model has recognized abnormalities, classifying them as COVID-19.

It is realized that patterns of other statements or ailments are not seen and learned throughout the training procedure. They also appear in the same areas of the lungs as COVID-19. Using this created model, they may readily be fooled by COVID-19. It may result in many false positive instances, which might not be desirable for real situations. As a result, the model is improved by using example chest X-ray pictures with more notes and illnesses in the training and verifying procedures. It allows the model to learn to distinguish between COVID-19 patterns and patterns from other disorders. Consequently, the specificity score of COVID-19 is increased while the false detection rate is reduced.

The generated model is then trained to categorize a chest X-ray image into three COVID-19 normal and other illness groups. It might keep the sensitivity score stable while increasing the specificity score because separating the non-COVID-19 class from the class of different diseases might prevent misunderstandings between COVID-19 and other conditions and uncertainty between normal and other illnesses. It is experimentally observed that the individual deep learning model cannot perform equally for all scenarios in terms of accuracy, precession, recall, specificity AUC, and F1-score; therefore, adding visual interpretation of the results will help humans make better diagnosis decisions from indicated features and patterns on the output images.

**Figure 20:**
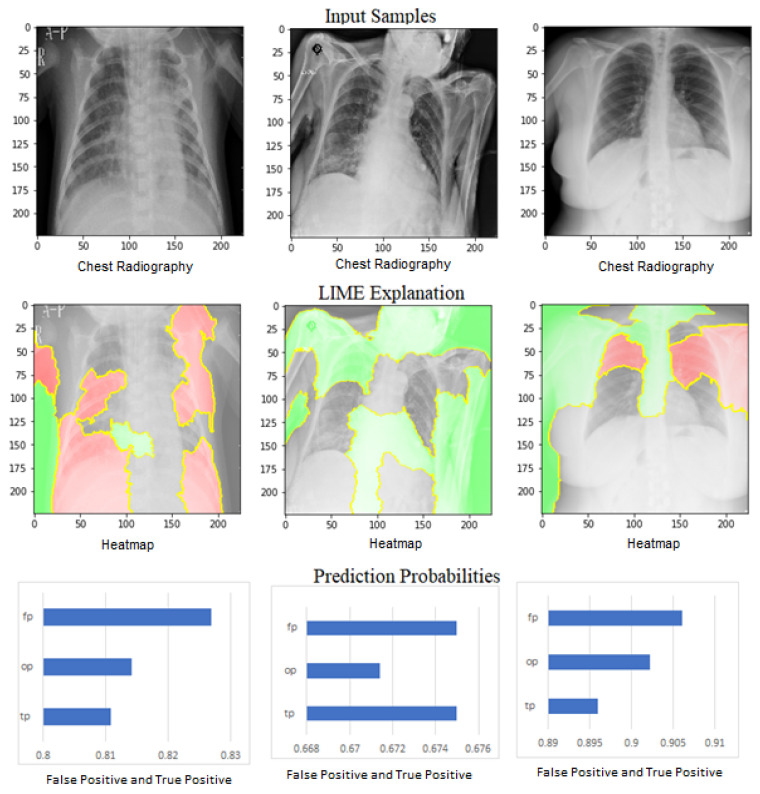
LIME explanation of three class samples along with the prediction probabilities of each sample.

Figure 20 shows the LIME technique applied to three samples belonging to three distinct classes of COVID-19. The red and green areas in the LIME-generated explanation correspond to the regions that contributed against and toward the predicted class, respectively.

### Architecture Comparison

In this section, we compare the performance of the proposed deep neural network architecture for detecting COVID-19 from chest CT images with existing architectures in terms of test data. Specifically, we compare it with Xception, Inception V4, ResNet-50, XNet, and AlexNet (deep learning architectures). Table 7 shows that VGG16 with LIME achieves a 0.2% higher test accuracy than Xception, using 90.6% fewer parameters. Additionally, Table 7 demonstrates that VGG16 has higher precision and recall than Inception V4 across all types of infections. Moreover, using VGG 16 also increases the performance of ResNet50, XNet, and AlexNet with accuracy improvement of 0.50%, 0.16, and 0.48, respectively. These findings emphasize the advantages of using VGG 16 design with LIME exploration to develop deep neural network architectures that are explainable to the task, data, and operational requirements. This is especially important in clinical settings, where quickly creating and assessing new architectures is crucial for adapting to changing data patterns and operational needs.

**Figure 21:**
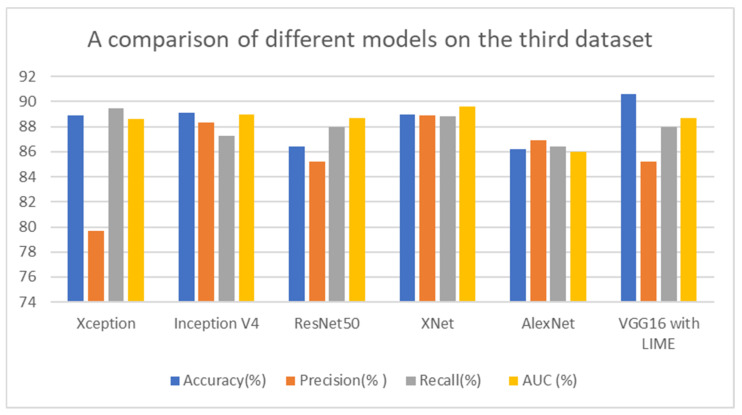
A comparison of different models on the third dataset.

As shown by Table 7, accuracy represents the overall correctness of the model’s predictions, while precision measures the proportion of true positive predictions among all positive predictions. Recall measures the proportion of true positive predictions among all actual positive instances in the dataset. The AUC metric represents the model’s ability to distinguish between positive and negative instances. Computation cost of each model is also presented by the table to show the difference in cost over models on different datasets. The metrics show that VGG16 with LIME has the highest overall performance, with 90.6% accuracy, precision, recall, and AUC. However, the performance difference between VGG16 with LIME and the other models is relatively small, with most models achieving an accuracy above 86%, as shown by Figure 21. The accuracy of datasets four and five is high because the dataset size is very small with 317 images and 97 images, respectively.

**Figure 22:**
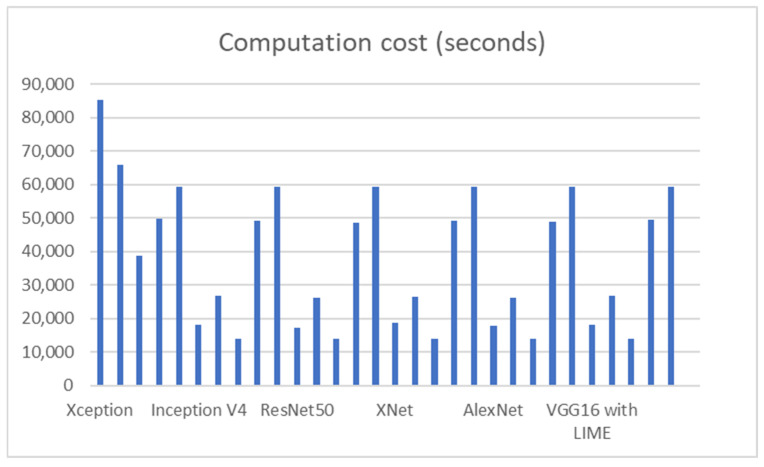
Computation costs of different models in seconds.

For testing performance measurement, we rely on dataset three because it is the largest dataset, and the testing accuracy shows that the model performs very well compared to other models. The reliance on dataset three for testing performance measurement was driven by its size and significance. Our model’s exceptional testing accuracy further reinforces its robustness compared to other models. By subjecting the model to diverse datasets during testing, we provided substantial evidence supporting its generalization capabilities and its resilience against overfitting. Moreover, Figure 22 shows that our model with LIME presented the smallest computational cost which is 10,936 s on the third dataset.

## 5. Conclusions and Remarks

This article proposes using deep learning models to aid in diagnosing the COVID-19 virus by using chest X-ray images with visual representation based on a local interpretable model. Prediction explanations of the model improve the final human decision. The model’s performance (regarding time, complexity, and accuracy) improved, obtaining more explainable results. The accuracy achieved was 90.6% on a relatively small size dataset. First, this model’s main advantages are the XAI model, which improves the interpretability and explainability of AI models. The experiment has been conducted using five COVID-19 datasets from Kaggle.com. The second advantage is the improvement of the model’s accuracy and explainability. The highest levels of precision we achieved were 90%, 93%, and 95%. The results show that VGG16 with LIME has the highest overall performance, with 90.6% accuracy, precision, recall, and AUC. However, the performance difference between VGG16 with LIME and the other models is relatively small, with most models achieving an accuracy above 86%. Furthermore, using our approach for comparable COVID-19 datasets to obtain further insights into crucial characteristics connected to COVID-19 instances will be beneficial.

Regarding the achieved accuracy on a relatively small dataset of 6432 images, it is important to note that several factors, including the complexity of the task, dataset size, and diversity, influence accuracy. However, our proposed model demonstrated a commendable accuracy of 90.6% on this dataset, which is a significant improvement compared to previous approaches. Moreover, our model improved accuracy and reduced time complexity, making it more practical and efficient for medical diagnosis in real-world scenarios. This reduction in time complexity allows for quicker decision-making, enabling prompt and accurate diagnoses.

Furthermore, our approach emphasizes interpretability and transparency in AI models, which are crucial aspects of the medical domain. By providing clear and understandable explanations for the model’s decisions, clinicians and healthcare professionals can better comprehend the underlying reasoning and build trust in the system. This interpretability enhances accuracy and facilitates collaboration between AI and human experts, improving overall diagnostic outcomes.

This research, however, is subject to several limitations. The first is the method compatible with the image dataset, which is challenging to use with the numerical dataset. Moreover, the empirical results reported here should be considered in light of some limitations like low quality or imbalanced data. In addition, working with medical data from the early stages of illness, such as COVID-19, has several drawbacks, including the dataset size. As new data becomes available, further models of COVID-19 infection may be developed. It is important to acknowledge the challenges that XAI as a field still faces in meeting the expectations of end-users, regulators, and the general public.

It is also difficult to objectively measure the accuracy of LIME explanations and determine whether they are right or wrong, as XAI explanations are subjective and dependent on human interpretation. While LIME is a widely accepted method, we acknowledged its limitations and focused on the availability and usefulness of the explanations. We validated the interpretability of LIME explanations with human experts, ensuring alignment with domain knowledge. Thus, while LIME contributed to our model’s explainability, evaluating the correctness of its explanations remains challenging, underscoring the need for further research in evaluating XAI model performance.

Our study focused on developing an accurate and interpretable XAI model for medical imaging classification. The training was conducted offline, but the real-time application or inference time was not directly impacted. Our emphasis was on evaluating the model’s performance and interpretability.

In the future, it is probable to find essential patterns in CT scans and utilize plots and charts to communicate predictions to patients. To this end, explaining these predictions in everyday language would be beneficial using a more human-interpretable evaluation method. The visual representation based on a classified interpretable model helps to reduce the diagnosis error using explanations of the model’s prediction. In addition, it can be used to give justifications behind decisions and evaluate all results. This model is important in Hybrid Deep Neural Networks (HDNNs), Computed Tomography, and Chest X-rays for detecting COVID-19.

## Figures and Tables

**Table 1:** A summary of related work.

Ref.	Proposed Model	Findings	Limitations
[7]	Deep CNN	Explainable predictions, deeper insights, making decisions based on relevant information	Densely connected deep neural networks increase computational complexity and memory cost.
[14]	Deep COVID Explainer	Augmented and classed using a neural ensemble technique with gradient-guided class activation maps (Grad-CAM++)	Accurate predictions are not solely based on single imaging modalities.
[4]	COVIDNet-CT	XAI-driven performance validation technique	Dependent on the availability of high-quality datasets
[10]	RcoNetk	Numerous parallel dropout networks to evaluate uncertainty	
[11]	RANDGAN	Segmented region of interest is critical for correctly learning the classification.	In some circumstances, the segmentation model fails.
[12]	Deep metric learning based CXR image retrieval model	Extract picture features from a new dataset	Semantic gap between information collected by computer algorithms and human perception
[19]	Unique multi-feature CNN	Demonstrated impact of improved pictures in enhancing diagnosis accuracy using ablation trials	AlexNet has roughly quadrupled the number of parameters with late fusion.
[17]	Ensemble learning with deep learning	Unique method for the evaluation of fuzzy measures	In some circumstances, it is impossible to calculate the marginal contribution
The Proposed Model	Deep CNN (VGG-16)	LIME generates a set of visualizations that can help clinicians better predict and identify potential sources of bias or error in the model’s reasoning.	LIME requires generating many perturbed samples around the original input to estimate the local feature importance weights. This can be computationally expensive in medical images.

**Table 2:** Division of the first dataset.

	Training	Testing	Validation	Total
Normal	1341	234	8	1583
Pneumonia	3875	390	8	4273
Total	5216	624	16	5856

**Table 3:** Division of the second dataset.

	Training	Testing	Total
Normal	1593	407	2000
Pneumonia	1107	273	1380
COVID-19	632	160	792
Total	3332	840	4172

**Table 4:** Division of the third dataset.

	Training	Testing	Total
Normal	1266	317	1583
Pneumonia	3418	855	4273
COVID-19	460	116	576
Total	5144	1288	6432

**Table 5:** Division of the fourth dataset.

	Training	Testing	Total
Normal	70	20	90
Pneumonia	70	20	90
COVID-19	111	26	137
Total	251	66	317

**Table 6:** Division of the fifth dataset.

	Training	Testing	Total
Normal	20	5	25
COVID-19	50	19	69
Total	70	24	94

**Table 7:** A comparison of different deep learning algorithms with the proposed model in testing data.

Architecture	Dataset	Accuracy (%)	Precision (%)	Recall (%)	AUC (%)	Computation Cost (s)
Xception	1	80.8	70.5	80.9	80.8	85,364
	2	65.9	56.7	66	66.1	65,972
	3	88.9	79.7	89.5	88.6	38,756
	4	94.8	85.6	95.9	94.5	49,856
	5	92.7	82.9	81.8	81.9	59,278
Inception V4	1	81.1	81.2	80.8	80.0	18,200
	2	67.2	68.1	68.1	68.0	26,781
	3	89.1	88.3	87.3	89.0	13,897
	4	94.1	92.2	91.9	91.1	49,129
	5	92.2	89.8	89.8	89.7	59,287
ResNet50	1	77.1	77.2	77.1	77.1	17,121
	2	63.4	64.2	63.6	63.2	26,342
	3	86.4	85.2	88.0	88.7	13,864
	4	89.5	90.1	89.7	89.3	48,645
	5	92.6	93.3	92.9	92.5	59,263
XNet	1	80.6	76.7	87.5	86.6	18,675
	2	65.5	87.3	87.3	87.0	26,587
	3	89.0	88.9	88.8	89.6	13,896
	4	94.1	91.1	91.2	91.1	49,121
	5	92.2	89.1	89.1	89.1	59,281
AlexNet	1	78.2	78.9	78.4	78.0	17,829
	2	63.2	63.9	63.4	63.0	26,324
	3	86.2	86.9	86.4	86.0	13,862
	4	89.2	89.9	89.4	89.0	48,928
	5	92.4	93.1	92.6	92.2	59,243
VGG16 with LIME	1	82.6	79.7	89.5	88.6	18,267
	2	67.5	88.3	87.3	89.0	26,758
	3	90.6	85.2	88.0	88.7	10,936
	4	95.7	88.9	88.8	89.6	49,578
	5	93.6	86.9	86.4	86.0	59,364

## Data Availability

Data associated with this research can be retrieved online as follows:
**Datasets****Link****Accessed On**Chest X-ray Images (Pneumonia)https://data.mendeley.com/datasets/rscbjbr9sj/219 December 2022COVID-19 And Pneumonia Chest X-rays Imageshttps://bimcv.cipf.es/bimcv-projects/bimcv-covid19/12 January 2023Chest X-ray Images (COVID-19 and Pneumonia)https://www.kaggle.com/datasets/prashant268/chest-xray-covid19-pneumonia22 January 2023COVID-19 Imagehttps://www.kaggle.com/datasets/pranavraikokte/covid19-image-dataset27 January 2023Chest X-ray (COVID-19 and Pneumonia)https://www.kaggle.com/datasets/alifrahman/covid19-chest-xray-image-dataset3 February 2023

## Appendix A. Datasets’ Validation Loss and Accuracy

**Table A1:** The five data-sets’ validation loss and accuracy reached ten epochs.

Results of the first dataset:		
Epoch	Validation loss	Validation accuracy
1	0.5377	0.6378
2	0.4627	0.7340
3	0.4332	0.7708
4	0.4231	0.7901
5	0.4122	0.8077
6	0.4048	0.8109
7	0.4026	0.8109
8	0.4019	0.8109
9	0.3980	0.8141
10	0.3904	0.8269
Results of the second dataset:		
Epoch	Validation loss	Validation accuracy
1	0.9360	0.5464
2	0.8680	0.6464
3	0.8290	0.6655
4	0.8051	0.6667
5	0.7860	0.6690
6	0.7751	0.6738
7	0.7644	0.6762
8	0.7561	0.6750
9	0.7491	0.6714
10	0.7412	0.6750
Results of the third dataset:		
Epoch	Validation loss	Validation accuracy
1	0.6315	0.6918
2	0.5001	0.8005
3	0.4270	0.8416
4	0.3801	0.8595
5	0.3476	0.8766
6	0.3238	0.8859
7	0.3052	0.8936
8	0.2907	0.8960
9	0.2781	0.9022
10	0.2678	0.9061
Results of the fourth dataset:		
Epoch	Validation loss	Validation accuracy
1	0.9912	0.6000
2	0.9695	0.6939
3	0.9412	0.7439
4	0.9169	0.7839
5	0.9002	0.8606
6	0.8906	0.8967
7	0.8040	0.9018
8	0.7790	0.9121
9	0.7546	0.9424
10	0.7322	0.9579
Results of the fifth dataset:		
Epoch	Validation loss	Validation accuracy
1	0.4355	0.6470
2	0.3617	0.7642
3	0.2322	0.7928
4	0.2231	0.8456
5	0.2182	0.8571
6	0.2047	0.8609
7	0.2026	0.8874
8	0.1961	0.8938
9	0.1880	0.9064
10	0.1746	0.9369
